# Understanding Vaccine Hesitancy: A Comparison of Sociodemographic and Socioeconomic Predictors with Health Literacy Dimensions

**DOI:** 10.3390/vaccines12101141

**Published:** 2024-10-04

**Authors:** Monika Lamot, Andrej Kirbiš

**Affiliations:** Department of Sociology, Faculty of Arts, University of Maribor, 2000 Maribor, Slovenia; monika.lamot1@um.si

**Keywords:** vaccine hesitancy, health literacy, sociodemographic factors, socioeconomic factors

## Abstract

Vaccine hesitancy represents a global public health challenge that can diminish the effectiveness of vaccination programs. Research indicates that various sociodemographic and socioeconomic factors, along with health literacy, predict vaccine hesitancy. In this study, we analyzed data from a Slovenian health literacy survey that included 3360 adult participants. We examined the effects of sociodemographic (gender and age) and socioeconomic factors (education, economic deprivation, and self-assessed socioeconomic status), as well as different dimensions of health literacy (general, communicative, and navigational), on vaccine hesitancy. The results show that age, education, and economic deprivation are statistically significant predictors of vaccine hesitancy; younger individuals, those with lower education levels, and those experiencing greater economic deprivation express increased vaccine hesitancy. Higher levels of general and communicative health literacy are associated with lower vaccine hesitancy, while navigational health literacy was not found to be a statistically significant predictor of vaccine hesitancy.

## 1. Theoretical Background

Vaccine hesitancy is a global public health challenge that can negatively impact the success of vaccination programs and the achievement of herd immunity [1]. It is a complex phenomenon that involves reservations, doubts, and fears about vaccination, despite the availability of safe and effective vaccines [2]. Vaccine hesitancy is often explained using the “3 Cs” model, which includes confidence, complacency, and convenience. Confidence refers to trust in vaccines, healthcare providers, and policymakers. Complacency occurs when individuals perceive the risk of vaccine-preventable diseases as low, reducing their perceived need for vaccination. Convenience involves the accessibility and affordability of vaccines, as well as the quality of services [2]. Understanding the factors that contribute to vaccine hesitancy is crucial for the development of strategies that promote greater acceptance of vaccination, thereby reducing the risk of the spread of infectious diseases.

Among the most frequently studied factors influencing vaccine hesitancy are sociodemographic and socioeconomic factors [3,4,5], as well as health literacy [6,7,8], which plays a key role in individuals’ ability to acquire, understand, and use health information [9,10]. In this study, we examine and compare the predictive power of sociodemographic factors (gender and age); socioeconomic factors (education, economic deprivation, and self-assessed socioeconomic status); and three dimensions of health literacy, namely general, communicative, and navigational health literacy, as predictors of vaccine hesitancy in Slovenia.

### 1.1. Relationship between Sociodemographic Factors and Vaccine Hesitancy

Previous studies have consistently shown that gender and age are statistically significant predictors of vaccine hesitancy [11,12,13]. Women, in particular, have been found to express higher levels of vaccine hesitancy than men [3,14,15]. This increased hesitancy among women is attributed to several factors, including heightened concerns about vaccine safety, a stronger preference for natural immunity, and a greater distrust in health institutions and pharmaceutical companies [16,17,18]. However, the effect of gender is not geographically consistent, as it was not found to be as a statistically significant predictor of attitudes toward vaccination in some countries [19]. In Slovenia, however, women have been found to be more vaccine-hesitant than men, regarding both COVID-19 vaccines [20] and vaccines in general, even before the pandemic [21].

**Hypothesis** **1:** *Women express higher vaccine hesitancy than men.*

Regarding age, younger individuals often express more vaccine hesitancy than older individuals [13,22,23,24,25]. Several factors contribute to this trend. One of the key reasons is that younger people tend to perceive themselves as being at lower risk of severe health outcomes from vaccine-preventable diseases, which may reduce their sense of urgency to get vaccinated. In addition, younger individuals are more likely to rely on social media as a primary source of information about vaccines, where they are frequently exposed to misinformation and conspiracy theories, and they may be more exposed to inaccurate information about vaccines [13].

**Hypothesis** **2:** *Younger individuals express higher vaccine hesitancy than older individuals.*

### 1.2. Relationship between Socioeconomic Factors and Vaccine Hesitancy

Socioeconomic factors such as education, economic deprivation, and self-assessed socioeconomic status have been found to significantly predict attitudes toward vaccination. Generally, higher levels of education and socioeconomic status are associated with more positive attitudes toward vaccination [26,27,28], as individuals with higher socioeconomic status and educational attainment tend to place more trust in vaccines and recognize their importance for public health [29]. However, the relationship between education, socioeconomic status, and vaccine hesitancy is not always straightforward. While many studies support the positive association, as noted above, some research suggests that these factors may not consistently predict vaccine hesitancy. For instance, in a pre-pandemic Slovenian study conducted using a non-representative sample, Kirbiš [21] found that neither education nor socioeconomic status was a significant predictor of vaccine hesitancy, although higher income was associated with greater hesitancy. Conversely, Lamot et al. [20] reported that lower education was significantly linked to COVID-19 vaccine hesitancy in a different Slovenian study, although their study also relied on a non-representative sample. These conflicting findings highlight the complexity of the relationship between socioeconomic factors and vaccine hesitancy. In line with most studies, though, we propose the following hypothesis and research question:

**Hypothesis** **3:** *Higher self-assessed socioeconomic status negatively predicts vaccine hesitancy.*

**Research Question** **1:** *What is the effect of education on vaccine hesitancy in Slovenia?*

Economic deprivation, on the other hand, has been consistently linked to higher levels of vaccine hesitancy [30,31]. Individuals with lower incomes are often more vulnerable to misinformation surrounding vaccines [32], as financial insecurity can limit access to reliable health information and hinder critical engagement with scientific content. This susceptibility is further exacerbated by a general lack of trust in scientific institutions and healthcare systems, which is more prevalent among economically disadvantaged groups [33].

**Hypothesis** **4:** *Economic deprivation positively predicts vaccine hesitancy.*

### 1.3. Relationship between Health Literacy and Vaccine Hesitancy

Numerous studies indicate that individuals with higher health literacy—defined as the ability to obtain, understand, and use basic health information to make informed health decisions—tend to have more positive attitudes toward vaccination and express less vaccine hesitancy [6,7,8,34]. This relationship was further confirmed by a systematic review that included thirteen studies and demonstrated that higher health literacy consistently predicts lower vaccine hesitancy [7]. A study conducted by Turhan et al. [8] also showed that hesitancy toward COVID-19 vaccination was associated with low health literacy and high levels of distrust in the healthcare system. Notably, in this study, health literacy acted as a mediator, serving as an intermediary in the relationship between distrust in the healthcare system and vaccination hesitancy [8]. Since the relationship between general health literacy and vaccine hesitancy appears to be consistent across multiple studies, we propose the following hypothesis:

**Hypothesis** **5:** *Health literacy negatively predicts vaccine hesitancy.*

On the other hand, communicative health literacy—which includes the ability to effectively search for and understand health information obtained through various communication channels—has been reported to negatively predict vaccination, as noted by Aharon et al. [35]. Navigational health literacy, another dimensions of heath literacy, refers to the ability to access and utilize healthcare services effectively, including knowing how to find appropriate care, follow healthcare processes, and use available resources [36]; however, to the best of our knowledge, the relationship between navigational health literacy and vaccination has not yet been studied.

**Hypothesis** **6:** *Communicative health literacy positively predicts vaccine hesitancy.*

**Research Question** **2:** *What is the effect of navigational health literacy on vaccine hesitancy?*

## 2. Methods

### 2.1. Sample

We analyzed data from the Health Literacy Survey of Adults in Slovenia (HLS-SI19) [37], which was part of the M-POHL research initiative. Data were collected using a combination of computer-assisted personal interviews (CAPIs), self-administered questionnaires (SAQs), and computer-assisted web-based interviews (CAWIs). Participants were selected based on a multi-stage random sampling procedure to ensure representativeness across various demographic categories, including age, gender, and geographical region. Inclusion criteria required that participant be permanent residents aged 18 and above. The survey was conducted in 2020 during the COVID-19 pandemic and included 3360 participants, of whom 53.7% were women. Participant age ranged from 18 to 99 years (M = 51.6). Most participants reported having a vocational high school education (25.3%), lower or middle vocational education (17.8%), or primary education (12.8%), followed by those with general secondary education (11.4%) and a second-level higher education (11.4%). A proportion of 6.6% reported having first-level higher education, and 9.2% had a higher professional education. Regarding employment status, most participants were employed (46.3%), followed by retirees (34.1%), unemployed (6.3%), self-employed (5.0%), and those still in education (4.9%).

### 2.2. Measurement Instruments

#### 2.2.1. Vaccine Hesitancy

Vaccine hesitancy was measured the following four statements: “Vaccination is important to protect me and my children”; “I generally believe that vaccinations are safe”; “I generally believe that vaccinations are effective”; and “Vaccination is important to prevent the spread of (serious) diseases”. Participants responded using a four-point scale (1 = strongly agree, 2 = agree, 3 = disagree, and 4 = strongly disagree) [37]. The final vaccine hesitancy score was calculated by averaging participants’ responses across all four items, resulting in a mean score ranging from 1 to 4, with higher scores indicating higher hesitancy. The scale shows good internal reliability (Cronbach’s alpha = 0.91).

#### 2.2.2. Health Literacy

Health literacy was assessed using the HLS-EU-Q47 questionnaire, translated into Slovenian [37]. The questionnaire includes 47 statements, whose difficulty was rated by the respondents. The questionnaire covers three dimensions or factors, namely health care, disease prevention, and health promotion, with each factor further divided into the following four dimensions: accessing or acquiring health information, understanding health information, appraising health information, and using health information. Examples of statements include “Find information on how to act in medical emergencies”; “Judge when you need to see a doctor”; and “Understand information on food packaging”. Respondents answered using a four-point scale (1 = very difficult, 2 = difficult, 3 = easy, and 4 = very easy). The health literacy score was calculated by summing the responses across all items and converting the result to a 0-to-100 scale, where higher scores indicate higher health literacy [37]. The scale shows excellent internal reliability (Cronbach’s alpha = 0.97).

#### 2.2.3. Communicative Health Literacy

Communicative health literacy was assessed using eleven statements, e.g., “Do you describe to your doctor the reasons for your visit?”; “Do you express personal opinions and wishes to your doctor?”; and “Do you ask questions to your doctor during a consultation?”. Participants responded using a four-point scale (1 = very difficult, 2 = difficult, 3 = easy, and 4 = very easy) [37]. The statements were aggregated into a single variable in the same manner as the health literacy questionnaire, resulting in a combined variable ranging from 0 (low health literacy) to 100 (excellent health literacy). The construct shows excellent internal reliability (Cronbach’s alpha = 0.94).

#### 2.2.4. Navigational Health Literacy

We measured navigational health literacy with twelve statements, whose difficulty was rated using a four-point scale (1 = very difficult, 2 = difficult, 3 = easy, and 4 = very easy) [37]. Examples of statements include “Do you understand information about how the healthcare system works (e.g., what types of healthcare services are available)?”; “Can you find information about the quality of a particular healthcare service?”; and “Can you obtain information about different options that can help you navigate the healthcare system more easily?” Navigational health literacy is aggregated in the same way as health literacy and communicative health literacy and shows excellent internal reliability (Cronbach’s alpha = 0.93).

#### 2.2.5. Sociodemographic and Socioeconomic Predictors

We examined sociodemographic predictors, including gender (1 = male and 2 = female) and age (entered in years). Among socioeconomic variables, we included education (1 = no formal education and 11 = master’s/doctorate), economic deprivation (measured by the statement, “How easy or difficult is it for you financially to pay bills for living expenses every month?” (1 = very easy; 4 = very difficult)), and self-assessed socioeconomic status (1 = lowest socioeconomic level in society; 10 = highest socioeconomic level in society).

### 2.3. Statistical Analyses

Statistical analyses were performed using SPSS 29.0 software. First, we calculated descriptive statistics (M and SD), followed by bivariate analyses, including Pearson correlations, ANOVA, and independent-sample *t*-tests. In the second part, we performed multivariate analysis using hierarchical linear regression, which was conducted in three steps. In the first step, sociodemographic variables (age and gender) were included in the model. In the second step, socioeconomic variables (education, economic deprivation, and self-assessed socioeconomic status) were added. In the final step, health literacy variables, including general health literacy, communicative health literacy, and navigational health literacy, were included in the model. This step allowed us to assess the additional predictive power of these variables with respect to vaccine hesitancy. The effectiveness of the hierarchical regression model was evaluated by examining changes in explained variance at each step and the statistical significance of individual predictors.

## 3. Results

### 3.1. Bivariate Analysis

Table 1 presents the descriptive statistics and the results of correlation analysis. In our sample, participants exhibited low vaccine hesitancy (M = 1.82; SD = 0.58) and demonstrated sufficient general health literacy (M = 68.17; SD = 14.36), as well as sufficient communicative health literacy (M = 74.74; SD = 16.39). However, the sample revealed problematic levels of navigational health literacy (M = 58.65; SD = 18.65), indicating difficulties in accessing and understanding information across health contexts. We also found that vaccine hesitancy is negatively associated with health literacy (r = −0.26; *p* < 0.01), communicative health literacy (r = −0.24; *p* < 0.01), and navigational health literacy (r = −0.19; *p* < 0.01), suggesting that health literacy decreases vaccination hesitancy, regardless of the measured dimension. Health literacy is significantly positively correlated with socioeconomic status (r = 0.28; *p* < 0.01) and negatively correlated with economic deprivation (r = −0.29; *p* < 0.01).

Furthermore, we examined the differences in gender and education in vaccine hesitancy. Table 2 shows the results of an independent-sample *t*-test, which confirmed the result of the univariate analysis, indicating no statistically significant differences in vaccine hesitancy between men and women (t (3080.40) = 0.115, *p* = 0.909).

An ANOVA was conducted to examine the differences in vaccine hesitancy across various levels of educational attainment (Table 3). The analysis revealed significant differences between multiple educational groups (*F* (10, 3087) = 11.174, *p* < 0.001), with a small effect size (η² = 0.03). Individuals with no formal education reported the highest vaccine hesitancy (M = 2.44, SD = 1.27), significantly differing from several other groups. For instance, those with primary education (M = 1.91, SD = 0.57) and incomplete primary education (M = 1.93, SD = 0.52) showed significantly lower levels of hesitancy than those with no formal education. Master’s or PhD holders (M = 1.56, SD = 0.55) and those with specialized education (M = 1.40, SD = 0.44) demonstrated the lowest vaccine hesitancy, significantly differing from the majority of lower-education groups, including secondary technical education (M = 1.87, SD = 0.56), indicating that individuals with higher education tend to exhibit lower vaccine hesitancy overall. In particular, the difference between individuals with specialization degrees (M = 1.40, SD = 0.44) and those with no formal education (M = 2.44, SD = 1.27) is substantial, reflecting a potential gradient where higher educational attainment correlates with reduced hesitancy.

Overall, the results suggest that higher levels of education, particularly specialized or postgraduate degrees, are associated with significantly lower vaccine hesitancy, while those with little to no formal education tend to report higher levels of hesitancy.

### 3.2. Multivariate Analysis

Next, we examined the predictors of vaccine hesitancy (Table 4). Hierarchical regression showed that in Model 1, gender and age were not statistically significant predictors of vaccine hesitancy (R² = 0.00, F = 0.52, *p* > 0.05). In Model 2, when socioeconomic predictors were included, education (β = −0.12, *p* < 0.001) and economic deprivation (β = 0.14, *p* < 0.001) were found to be statistically significant predictors of vaccine hesitancy, with those with higher education levels expressing lower vaccine hesitancy and those reporting higher economic deprivation expressing increased vaccine hesitancy. Self-assessed socioeconomic status was not a statistically significant predictor of vaccine hesitancy.

In Model 3, when indicators of health literacy (general health literacy, communicative health literacy, and navigational health literacy) were added, an additional 5% of the variance in understanding vaccine hesitancy was explained (∆R² = 0.05; ∆F = 57.15; *p* < 0.001). Health literacy (β = −0.17; *p* < 0.001) and communicative health literacy (β = −0.13; *p* < 0.001) were statistically significant negative predictors of vaccine hesitancy, while navigational health literacy was not a statistically significant predictor. It was also found that age (β = −0.07; *p* < 0.001), education (β = −0.09; *p* < 0.001), and economic deprivation (β = 0.09; *p* < 0.001) statistically significantly predict vaccine hesitancy. Specifically, those with lower levels of both general and communicative health literacy, younger individuals, those with lower education, and those reporting higher economic deprivation expressed more vaccine hesitancy. Based on the results, hypotheses H2, H4, and H5 received support, whereas hypotheses H1 and H3 were not supported, as gender and socioeconomic status did not significantly predict vaccine hesitancy.

## 4. Discussion

The results of this study indicate that sociodemographic and socioeconomic factors, such as gender, age, education, economic deprivation, and health literacy, significantly predict vaccine hesitancy. Younger individuals expressed more hesitancy, while higher education and better general and communicative health literacy reduce vaccine hesitancy. Conversely, economic deprivation increases vaccine hesitancy. Navigational health literacy did not have a statistically significant effect on vaccine hesitancy.

The study’s findings align with those of previous studies that examined the impact of sociodemographic and socioeconomic factors on vaccine hesitancy. Specifically, the findings are consistent with studies showing that higher education and lower economic deprivation negatively predict vaccine hesitancy [3,5,30,31] and that younger individuals show more hesitancy toward vaccination [11,12,13]. Regarding health literacy, our findings align with studies indicating that higher levels of health literacy reduce vaccine hesitancy [6,7,8]. Interestingly, contrary to the study conducted by Aharon et al. [35], which reported a negative effect of communicative health literacy on attitudes toward vaccination, our research indicates that this type of literacy has a positive effect in Slovenia. This discrepancy may be attributed to differences in how communicative health literacy is conceptualized and measured in the two studies. In the study by Aharon et al. [35], communicative health literacy was assessed in the specific context of parents’ ability to search for and comprehend vaccination-related information for their children. The measurement items primarily focused on the respondents’ ability to proactively gather information from a wide range of sources, including the Internet, media, and social networks. This broad and self-directed information-seeking process may have inadvertently exposed respondents to misinformation or contradictory content, which could have contributed to heightened vaccine hesitancy. Given the complexity and volume of information available on vaccination, potential exposure to misleading information could increase confusion and amplify concerns about vaccine safety.

In contrast, the present study conceptualized communicative health literacy as individuals’ ability to communicate effectively with healthcare professionals. The focus was on participants’ capacity to explain their health concerns, ask relevant questions, and fully understand the information provided by their doctors. This approach emphasizes the quality of doctor–patient communication, where individuals can engage in direct, meaningful exchanges with a trusted healthcare provider. The findings suggest that individuals with higher communicative health literacy are not only better-equipped to articulate their concerns but also more likely to receive accurate and tailored medical advice, fostering confidence in their health-related decisions, including vaccination choices. The interpretation of the positive impact of communicative health literacy is two-fold. First, individuals with higher communicative health literacy are more likely to engage in productive interactions with their doctors, which can increase trust in medical recommendations—a key factor in reducing vaccine hesitancy [38]. Secondly, effective communication with healthcare professionals allows individuals to clarify any doubts or concerns they may have about vaccines, thereby reducing the potential influence of misinformation. Misinformation is a well-established predictor of vaccine hesitancy [39], and the ability to seek and receive reliable information from trusted sources likely mitigates the effects of misleading or false information about vaccines. By fostering open dialogue with their doctors, individuals are better able to resolve uncertainties and make informed decisions, leading to more positive attitudes toward vaccination. This highlights the important role of healthcare providers as gatekeepers of accurate information, particularly in an era in which misinformation is pervasive.

The findings of the present study show that general and communicative health literacy had the largest effect sizes, indicating their key roles in shaping attitudes toward vaccination. General health literacy equips individuals with the ability to evaluate and interpret health information, enabling informed decision making. Communicative health literacy, on the other hand, enhances individuals’ capacity to engage with healthcare professionals and seek clarification when needed [37]. Together, these skills are essential in reducing vaccine hesitancy, as they not only ensure that people have access to accurate information but also empower them to communicate effectively about their health concerns. This highlights the importance of promoting health literacy at both the individual and community levels to foster informed, confident health decisions.

Interestingly, education and economic deprivation exhibited similar effect sizes, following health literacy. This suggests that socioeconomic factors such as an individual’s educational and financial background should not be overlooked when addressing vaccine hesitancy. Several studies have shown that higher education levels are associated with more positive attitudes toward vaccination, while lower education is linked to greater hesitancy [20,27]. Education affects not only an individual’s ability to critically assess health information but also their choice of information sources. As Roshchina et al. [40] noted, individuals with lower education levels are more susceptible to vaccine rumors and less willing to get vaccinated. This highlights the role of education in promoting critical thinking and the filtering out of misinformation.

Economic deprivation also plays a significant role in vaccine hesitancy. Individuals facing economic hardship are often more susceptible to inaccurate information about vaccines [32] and tend to exhibit lower trust in science [33]. Our findings show that individuals with higher economic deprivation exhibited lower levels of general, communicative, and navigational health literacy, further supporting the notion that economic barriers reduce the capacity to engage with and understand health information. The combination of low health literacy and distrust in science exacerbates vaccine hesitancy, making it crucial to address both educational and economic factors when designing interventions to increase vaccine uptake.

Lastly, younger age was associated with higher levels of vaccine hesitancy, which may be attributed to younger individuals perceiving themselves as being at lower risk of diseases such as COVID-19. This belief may lead younger individuals to feel that they do not need vaccines and contribute to lower overall trust in vaccines [41]. Such attitudes likely shape their broader perceptions of vaccination, contributing to increased hesitancy beyond just COVID-19. Additionally, greater exposure to misinformation through social media platforms, which are widely used by members of younger demographic groups, may further amplify concerns about vaccines [13].

There are some limitations of this study that should be acknowledged. First, the data were derived from a cross-sectional survey, which limits the ability to determine causality. Additionally, the study is based on participants’ self-assessments, which can lead to response bias. Furthermore, the sample is limited to the adult population in Slovenia, which may limit the generalizability of the results to other geographical and cultural contexts. Finally, the timing of the survey, conducted during the COVID-19 pandemic, may have influenced participants’ responses and attitudes, thereby limiting the applicability of the results to non-pandemic settings.

Based on the findings of this study, several actionable recommendations can be made to address vaccine hesitancy. First, targeted health literacy programs should be developed and implemented, particularly for individuals with lower education levels and those in vulnerable socioeconomic positions. These programs should not only provide accurate health information but also focus on improving individuals’ abilities to access, evaluate, and apply this information. Both general and communicative health literacy emerged as key factors in reducing vaccine hesitancy, highlighting the need for interventions that empower people to make informed decisions and engage effectively with healthcare providers.

Secondly, tailored communication strategies for younger individuals are essential. Since younger populations exhibited higher levels of vaccine hesitancy, likely due to lower perceived risk of disease and increased exposure to misinformation through social media, communication strategies should be adapted to resonate with these groups. Public health campaigns can leverage social media platforms and influencers to counter misinformation and promote credible, evidence-based information in formats that are engaging and relevant to younger audiences. These campaigns should also emphasize the potential risks of vaccine-preventable diseases for younger individuals and the broader community.

Finally, healthcare providers play a pivotal role in addressing vaccine hesitancy through effective communication with patients. Training for healthcare professionals should focus on the importance of clear, empathetic dialogue when discussing vaccines. By fostering open conversations and addressing patients’ concerns directly, healthcare providers can build trust and help mitigate the impact of misinformation.

## 5. Conclusions

Vaccine hesitancy remains a critical public health challenge, with this study underscoring the importance of sociodemographic, socioeconomic, and health literacy factors in shaping individuals’ attitudes toward vaccination. Younger individuals showed higher vaccine hesitancy, whereas those with higher education and better general and communicative health literacy exhibited lower levels of hesitancy. Economic deprivation was linked to increased hesitancy, highlighting how financial hardship can limit access to reliable health information and foster distrust in healthcare systems. Interestingly, navigational health literacy was not a significant predictor, suggesting that other health literacy dimensions, particularly the ability to engage effectively with healthcare providers, may have greater influence.

## Figures and Tables

**Table 1:** Descriptive statistics and Pearson correlations between continuous variables.

	M	SD	1.	2.	3.	4.	5.	6.
1. Vaccine hesitancy	1.82	0.58	-					
2. Health literacy	68.17	14.36	−0.26 **	-				
3. Communicative health literacy	74.74	16.39	−0.24 **	0.57 **	-			
4. Navigational health literacy	58.65	18.65	−0.19 **	0.71 **	0.54 **	-		
5. Age	51.64	17.69	0.02	−0.27 **	−0.13 **	−0.19 **	-	
6. Economic deprivation	2.41	0.71	0.20 **	−0.29 **	−0.22 **	−0.30 **	0.08 **	-
7. SES	5.42	1.60	−0.15 **	0.28 **	0.21 **	0.27 **	−0.15 **	−0.56 **

Note: SES = socioeconomic status. ** *p* < 0.01.

**Table 2:** Independent-sample *t*-test for vaccine hesitancy by gender.

	Men		Women		t (3080.40)	*p*	Cohen’s *d*
	M	SD	M	SD			
Vaccine hesitancy	1.82	0.56	1.82	0.61	0.12	0.909	0.58

**Table 3:** Differences in educational attainment with respect to vaccine hesitancy (ANOVA).

	M	SD
No formal education ^1^	2.44	1.27
Incomplete primary education ^2^	1.93 ^(10, 11)^	0.52
Primary education ^3^	1.91 ^(9, 10, 11)^	0.57
Lower or secondary vocational education ^4^	1.93 ^(6–11)^	0.52
Secondary technical education ^5^	1.87 ^(9, 10, 11)^	0.56
Gymnasium ^6^	1.79 ^(4, 10, 11)^	0.61
Higher vocational education ^7^	1.78 ^(4, 10)^	0.62
Higher professional education, BA ^8^	1.77 ^(4, 10)^	0.65
Higher university education, MA ^9^	1.66 ^(3, 4, 5)^	0.6
Specialization ^10^	1.40 ^(2–8)^	0.44
Master’s of science or PhD ^11^	1.56 ^(2–6)^	0.55
F	11.174 ***	
η^2^	0.03	

Notes: *** *p* < 0.001. Due to the non-homogeneity of variances, Welch’s F is reported. Superscript numbers indicate significant pairwise comparisons between profiles based on post hoc tests.

**Table 4:** Hierarchical regression.

	Model 1		Model 2		Model 3	
	β	S. E.	β	S. E.	β	S. E.
Gender (female)	−0.00	0.02	−0.00	0.02	0.01	0.02
Age	0.02	0.01	−0.03	0.00	−0.07 ***	0.00
Education			−0.12 ***	0.01	−0.09 ***	0.00
Economic deprivation			0.14 ***	0.02	0.09 **	0.02
SES			−0.02	0.01	−0.01	0.01
HL					−0.17 ***	0.00
CHL					−0.13 ***	0.00
NHL					0.03	0.00
*R^2^*	0.00		0.05		0.10	
*F*	0.52		32.34 ***		42.78 ***	
*∆R^2^*	0.00		0.05		0.05	
*∆F*	0.52		53.54 ***		57.15 ***	

Notes: β = standardized beta coefficient; S. E. = standard error for the unstandardized beta; SES = socioeconomic status; HL = health literacy; CHL = communicative health literacy; NHL = navigational health literacy. ** *p* < 0.01; *** *p* < 0.001.

## Data Availability

The data are available upon reasonable request to the authors.
